# Hospitals and the Treatment of Children under the Insurance Bill

**Published:** 1911-06-10

**Authors:** 


### HOSPITALS AND THE TREATMENT OF CHILDREN UNDER THE INSURANCE BILL.

By A HOSPITAL PHYSICIAN.

Should the Insurance Bill become law it is proposed that those only should receive medical treatment who have reached the age of the worker. Children at first are to be excluded. Later, however, they also are to share in the benefits to be derived from the scheme. This opens up great possibilities. There seems to be no reason why the much-debated difficulties concerning the treatment of school children should not disappear. Yet at first how great a share the hospitals and how great a share the private practitioner is to take in this treatment may be difficult to adjust. There are some who think that the majority of patients, whether adults or children, who now attend the out-patient departments of hospitals should be treated by the private practitioner, and that only those cases which the practitioner thinks are suitable for the advice or treatment to be obtained at a hospital should go there. In other words, the hospital out-patient departments should be considered to be places where special opinions are given. The name consultative clinics might perhaps be given. No doubt if the local practitioner could afford the time he would meet his patient at the hospital out-patient department for the purpose of an exchange of views with the out-patient physician or surgeon. This would react beneficially on both the local practitioner and the out-patient physician and surgeon, and the patient also, without doubt, should be a gainer.

At the present time by far the greater number of the out-patients at hospitals attend for trivial ailments. The long period patients may sometimes be kept waiting at hospitals is occasionally complained of. This is not the fault of those whose duties it is to attend to the patients. Were the number of out-patients diminished by careful selection there need be no unreasonable time spent in waiting before attention is given.

This remark applies to adults as well as to children, but medical inspection of schools has considerably increased the number of children requiring hospital treatment. The cases recommended treatment or advice in the school, it is scarcely necessary to remark, are not always trivial, but there is a large number of cases who could be well dealt with by a private practitioner were the parents in a position to pay for treatment. This remark applies not merely to cases requiring medical treatment but to cases where some minor operation has been advised. Unless special arrangements have been made at hospitals to deal with the additional number of cases since medical school inspection has become general, it is impossible to perform the operation for the removal of adenoids upon more than a comparatively small percentage of those who apply for the operation to be performed. Yet although remuneration for treatment of children by the State would enable such an operation to be performed by the private practitioner, the question arises whether he would often be able to perform it in his own surgery or at the home of the child. The operation could undoubtedly be more efficiently performed at a centre where facilities are offered for the carrying out of minor operations. It seems that what have been called "school clinics" will need to be established; centres at which not only medical men can conduct treatment but where some children would benefit by a daily visit for ailments requiring the attention of a trained nurse.

It can scarcely be denied that the treatment of children all over the country at the present time leaves much to be desired. An efficient organisation to deal with them is necessary, it may be said absolutely necessary. Should any such organisation greatly diminish the number of patients attending out-patient departments of children's hospitals it does not follow that the usefulness of those departments need be diminished. As has been indicated, they may be increased. If they were to cease entirely to provide treatment they should be of considerable value as affording facilities for obtaining an opinion. Difficult cases of diagnosis of heart, lung, or of the nervous system might be brought them, and in surgery or in skin affections cases in which a second opinion would be desirable would also be numerous.

Yet whatever might become of the out-patient departments the wards of a hospital must long remain a refuge for the afflicted in time of illness, and the only place in which skilled surgical treatment, in cases of major importance, can be obtained for those whose homes and means do not permit of all the disturbance of the affairs of everyday life which a surgical operation involves when carried out in the home. Hospitals have been one of the greatest blessings to a community and have never been more of a blessing than at the present moment. Yet in spite of their wide sphere of usefulness the medical treatment of the poor presents features which do not reflect great credit upon what we consider to be our high type of civilisation. Medical inspection of school children helps to reveal the limitations of the hospitals and the shortcomings of our economy.

